# Lipidomic Signatures and Associated Transcriptomic Profiles of Clear Cell Renal Cell Carcinoma

**DOI:** 10.1038/srep28932

**Published:** 2016-06-30

**Authors:** Kosuke Saito, Eri Arai, Keiko Maekawa, Masaki Ishikawa, Hiroyuki Fujimoto, Ryo Taguchi, Kenji Matsumoto, Yae Kanai, Yoshiro Saito

**Affiliations:** 1 Division of Medical Safety Science, National Institute of Health Sciences, 1-18-1 Kamiyoga, Setagaya, Tokyo, Japan; 2 Division of Molecular Pathology, National Cancer Center Research Institute, 5-1-1 Tsukiji, Chuo, Tokyo, Japan; 3 Department of Pathology, Keio University School of Medicine, 35 Shinanomachi, Shinjuku, Tokyo, Japan; 4 Department of Urology, National Cancer Center Hospital, 5-1-1 Tsukiji, Chuo, Tokyo, Japan; 5 Department of Allergy and Clinical Immunology, National Research Institute for Child Health and Development, 2-10-1 Okura, Setagaya, Tokyo, Japan

## Abstract

Renal cell carcinoma (RCC) is the most common histological type of adult kidney cancer. In this study, we obtained lipidomic profiles of clear cell RCC (ccRCC), a major RCC subtype, by performing a lipidomic analysis of specimens of cancerous tissue and the surrounding normal renal cortex obtained from the same patients (*N* = 49). We also compared the lipidomic profiles with the lipogenic transcriptome of specimens of cancerous tissue and the surrounding normal renal cortex for an additional set of patient samples (*N* = 95). Overall, we detected 326 lipids, including phospholipids, sphingolipids, neutral lipids, and eicosanoids. The levels of more than 70% of the detected lipids were significantly different (*P* < 0.01, corrected by the false discovery rate). The cancerous tissue was distinguished by higher levels of ether-type phospholipids, cholesterol esters, and triacylglycerols, as well as by lower levels of phospholipids (except for phosphatidylcholines) and polyunsaturated fatty acids. Characteristic changes in the levels of mRNAs and metabolites suggested that the phosphatidylethanolamine (PE) synthesis pathway is suppressed in ccRCC and associated with cell proliferation. The present study represents the lipidomic profiles of ccRCC, which provides novel information about the metabolic changes in renal cancerous tissue and RCC pathophysiology.

Renal cell carcinoma (RCC) is the most common type of adult kidney cancer and originates in the proximal convoluted tubules^1^,^2^. Although early stage RCC is curable by nephrectomy, it still causes over 100,000 deaths each year worldwide^3^. Many patients are diagnosed with RCC metastasis from the kidney^4^. Recently, a mutation in the Von Hippel-Lindau tumor suppressor gene was proposed as a prognostic biomarker for RCC^5^,^6^. In addition, specific targeting molecules have been developed for the treatment of RCC, although their effectiveness for metastasized RCC is very limited^7^. Thus, the mechanisms underlying renal carcinogenesis should be further elucidated in order to develop more selective and powerful targeted therapies for RCC patients.

Metabolic changes, such as the Warburg effect, are observed in most cancer cells. Alterations in lipid metabolism have also emerged as characteristic biochemical signatures of cancer cells^8^,^9^. For example, the fatty acid composition of phospholipids was found to be altered in nonalcoholic steatohepatitis (NASH)-associated hepatocellular carcinoma, as higher levels of monounsaturated fatty acids (FAs) (such as 16:1 and 18:1 FA) and lower levels of saturated fatty acids (such as 16:0 and 22:0 FA) were identified in tumors in a corresponding genetic mouse model^10^. In addition, phosphatidylethanolamine (PE) levels were found to be lower in MC4-L5 mammalian breast cancer cells than in mammalian epithelial cells (EpH4)^11^. Higher levels of ether-type phosphatidylcholine (ePC) and phosphatidylinositol (PI) were detected in the migratory breast cancer cell line MDA-MB-231^12^. Lower concentrations of sphingomyelins (SMs) and higher specific PI (38:3 PI) levels were found to be prominent in non-small cell lung cancer^13^. With regard to RCC, abnormally high levels of cholesterol ester (ChE) have been observed in clear cell renal cell carcinoma (ccRCC)^14^. In addition, triacylglycerol (TG) levels were also found to be higher in RCC^15^,^16^. However, overall knowledge about lipidomic alterations on the progression of human RCC remains very limited.

In the present study, we used the lipidomic approach, an emerging method used to obtain general lipid profiles, to determine the overall lipidomic alterations in human RCC. Lipid levels in the cancerous and normal tissues from patients with ccRCC, the major RCC subtype, were compared. We employed a recently developed liquid chromatography-mass spectrometry-based lipidomics platform, which combined a non-targeted approach for phospholipids, sphingolipids, neutral lipids, and acylcarnitines with a targeted approach for polyunsaturated fatty acids (PUFAs) and their metabolites^17^,^18^. In total, we were able to detect 326 lipids and showed that the levels of more than 70% of them were significantly different (false discovery rate <0.01) between the cancerous and normal tissues, with 46% and 27% of the lipids present at higher or lower levels in the pathological samples, respectively. These observations point to drastic alterations in the lipid metabolism in ccRCC. In addition, the observed changes in the lipid metabolism were correlated with the expression levels of lipogenic genes in ccRCC. Moreover, using RCC-derived cultured cells, we obtained further experimental data to support a possible role for lower PE levels in the proliferation of ccRCC cells. In summary, we present novel information about the lipidomic signatures in ccRCC and provide useful information concerning ccRCC biochemical mechanisms, which could be targeted by future therapeutic approaches.

## Results

### Global lipidomic profiles of ccRCC

Global lipidomic profiling using our assay platform helped detect 326 lipid molecules in the ccRCC samples (Table 1). These lipid molecules included phospholipids (117 molecules), sphingolipids (47 molecules), neutral lipids (112 molecules), PUFAs and their metabolites (38 molecules), and acylcarnitines (12 molecules). The major phospholipid classes, PCs and PEs, comprised 25 lipid molecules each. The PCs included 16:0/18:1 PC and 18:0/18:1 PC (Supplementary Table S1), which are agonists of peroxisome proliferator-activated receptor alpha (PPARα) in liver and muscle, respectively^19^,^20^. In addition, along with SMs and ceramides (Cers), our platform detected sulfatides (Suls), which are brain- and kidney-enriched ceramide derivatives. The TG class contained the largest number of lipids (76 molecules). Moreover, the PUFAs included arachidonic acid (20:4), eicosapentaenoic acid (20:5), and docosahexaenoic acid (22:6), which are major upstream substrates of prostaglandins and leukotrienes^21^,^22^. PUFA metabolites were three downstream products of the cyclooxygenase (COX), lipoxygenase (LOX), and cytochrome P450 (P450) enzymes.

**Figure 1:**
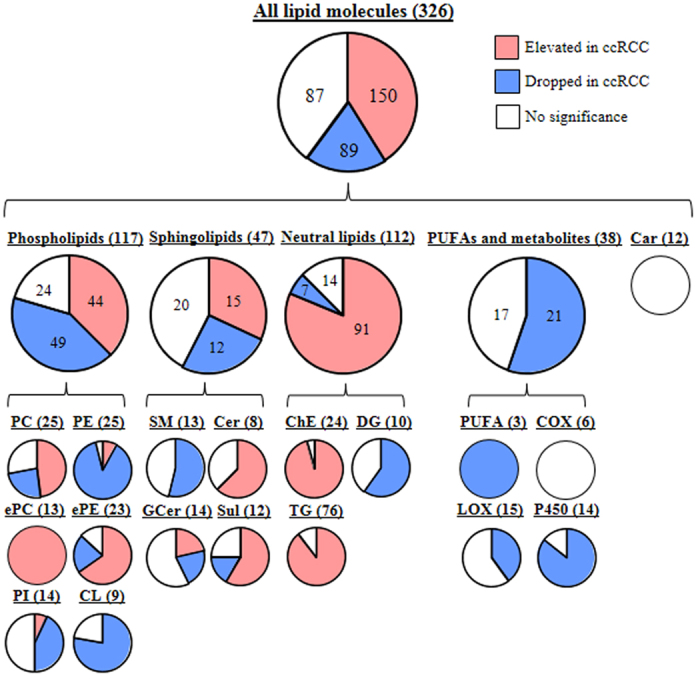
Diagram of the individual lipid molecules found at significantly different levels in the normal and ccRCC tissue samples. The numbers after the lipid class indicate the total number of lipid molecules in each lipid class. The numbers within the circle indicate the number of lipid molecules within the indicated category. The abbreviations are defined in Table 1.

To investigate the global lipid profiles, we used *P* < 0.01 (corrected with the false discovery rate [FDR]) as the threshold values of statistical significance in the present study. Overall, the levels of 239 lipids (73% of the total number of identified lipid molecules) were significantly different between the cancerous and normal tissues (Fig. 1). One hundred and fifty lipid molecules (46% of the total number identified) were present in higher levels in the ccRCC tissue, whereas the levels of 89 lipids (27% of the total number identified) were lower in the cancerous samples. The numbers of lipid molecules detected at higher and lower levels in the pathological tissue were comparable among phospholipids (44 vs 49) and sphingolipids (15 vs 12). On the other hand, neutral lipids were predominantly found at higher (91) rather than lower levels (7) in the cancerous tissue, whereas the levels of 21 PUFA molecules and their metabolites were lower in the pathological samples. Considering all lipid classes, we observed that the molecules that were frequently detected at higher levels in the ccRCC tissue included the PC, ePC, ether-type PE (ePE), Cer, Sul, ChE, and TG classes (48%, 100%, 65%, 63%, 58%, 96%, and 89% of the total number of molecules within their class, respectively). On the other hand, lipids found in lower levels in the cancerous tissue were predominantly PE, PI, cardiolipin (CL), SM, diacylglycerol (DG), PUFA, LOX metabolites, and P450 metabolites (88%, 43%, 78%, 54%, 60%, 100%, 40%, and 86% out of all molecules within their class, respectively). No differences in the levels of acylcarnitine or COX metabolites were observed between the normal and cancerous tissues. When we focused on the arachidonic acid metabolic pathway, we found that P450 and LOX metabolites were present at lower concentrations in the cancerous tissue, whereas the levels of COX metabolites were unaffected by pathological changes.

### Lipidomic network in ccRCC

**Figure 2:**
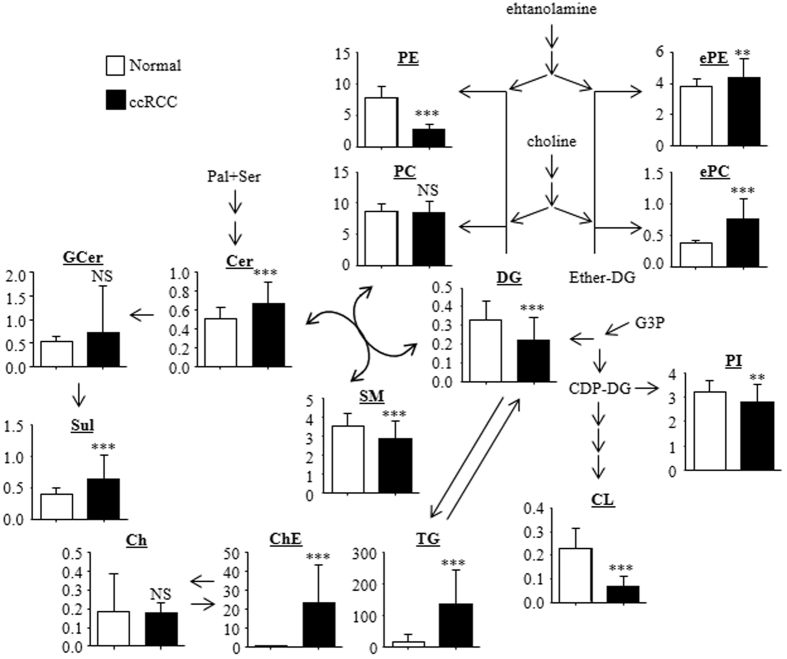
Lipidomic network in ccRCC. The data represent the sums of the ion peak heights of all lipid molecules within each class and are illustrated as the means ± SD (*N* = 49 in each group). The statistical significance of the differences between measurements in the cancerous and normal tissues was assessed using a paired Student’s *t*-test with FDR adjustment as follows: **P < 0.01; ***P < 0.001; NS, not statistically significant. The abbreviations are defined in Table 1.

For a further analysis of ccRCC lipidomic profiles, we constructed a lipidomic network pathway. Because the alteration patterns of the major lipid molecules within each lipid class showed a similar trend, we employed the summed levels of each lipid class to construct the lipidomic network (Fig. 2). As expected, the differences in the total levels of lipids from each lipid class (with the exception of PCs) were associated with the percentage of lipid molecules that demonstrated significantly altered levels between the cancerous and normal tissues (Fig. 1). The total levels of ePC, ePE, Cer, Sul, ChE, and TG molecules were higher, whereas the levels of PE, PI, CL, SM, and DG were lower in the ccRCC samples. The total levels of the PC lipids were comparable between the normal and cancerous samples, although 48% of the PC molecules were present in significantly higher levels in the pathological tissue.

**Figure 3:**
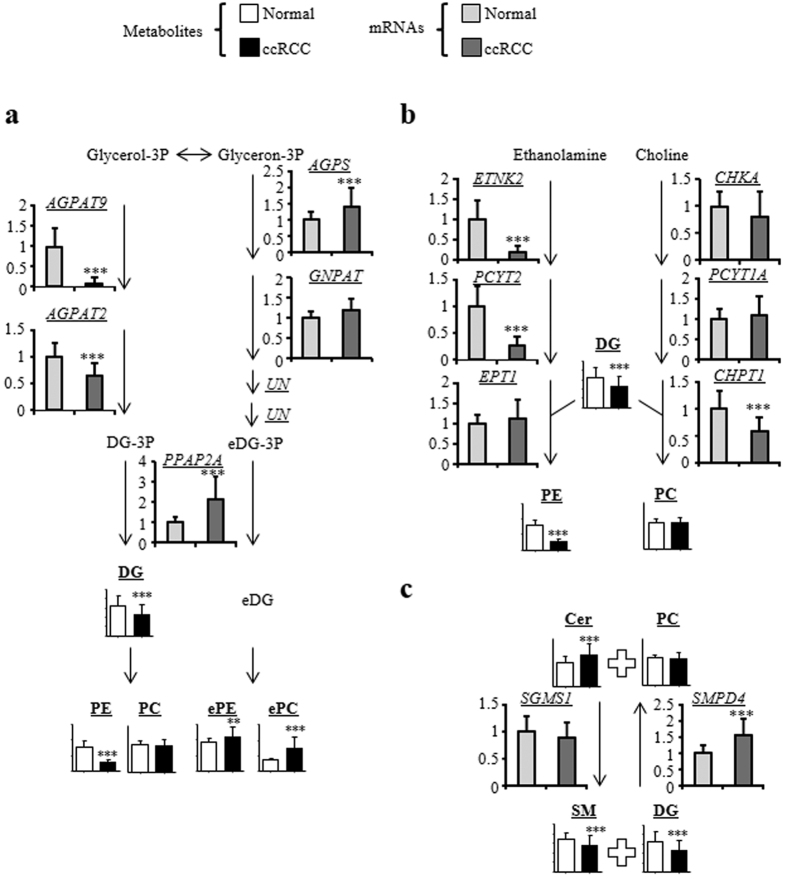
Levels of lipogenic gene mRNAs in highlighted lipid biosynthetic pathways. The gene with the highest average intensity was selected as the representative of each pathway. The data are presented as relative mean levels ± SD (*N* = 95 in each group), where the levels in the normal tissues were set to 1. The statistical significance of the differences between measurements in the cancerous and normal tissues was assessed using a paired Student’s *t*-test with FDR adjustment as follows: ***P < 0.001; NS, not statistically significant. The abbreviations are defined in Table 1 and Supplementary Table S5.

The drastic increase we saw in the ChE and TG levels in ccRCC is consistent with previous findings^14^,^15^,^16^. Furthermore, our microarray analysis confirmed that the expression of *VLDLR* and *ANGPTL4* (Supplementary Fig. S1), putative key genes for ChE and TG accumulation, was upregulated, as *VLDLR* and *ANGPTL4* were previously shown to be expressed at higher levels in the ccRCC tissue^23^,^24^,^25^,^26^. In addition to the accumulation of ChE and TG, our experiments also highlighted ccRCC-associated alterations in several other lipidomic pathways, such as ether-type phospholipid synthesis, PE synthesis, and the Cer and PC to SM and DG conversion pathways. Thus, we next focused on the mRNA expression levels of lipidomic genes involved in these lipidomic pathways (Fig. 3). As shown in Fig. 3a, genes specific for DG synthesis, *AGPAT9* and *AGPAT2*, were expressed at lower levels in the ccRCC samples, whereas the level of *AGPS,* a gene specific for ether-type DG (eDG) synthesis, was significantly higher. In addition, the level of *PPAP2A*, a common gene for DG and eDG synthesis, was expressed at significantly higher levels in the cancerous samples. These observations suggest that DG synthesis is downregulated in ccRCC, and eDG synthesis is enhanced. With respect to the PE synthesis pathway, the levels of *ETNK2* and *PCYT2*, but not of *EPT1*, were reduced two-fold in the ccRCC samples compared with the normal tissue (Fig. 3b). Thus, PE synthesis could also be downregulated in ccRCC. Unlike the PE levels, the levels of PC lipids were comparable between the normal and cancerous tissues. Within the PC synthesis pathway, levels of *CHPT1* were reduced in the pathological samples, whereas levels of genes encoding two other relevant enzymes, *CHKA* and *PCYT1A*, were not altered. Upregulation of the *SMPD4* gene, which encodes the enzyme responsible for the production of Cer and PC from SM and DG, reflected the decreased levels of SM and DG and increased levels of Cer in ccRCC (Fig. 3c). Thus, these alterations would lead to conserved PC levels in ccRCC.

### Fatty acid composition in PC

**Figure 4:**
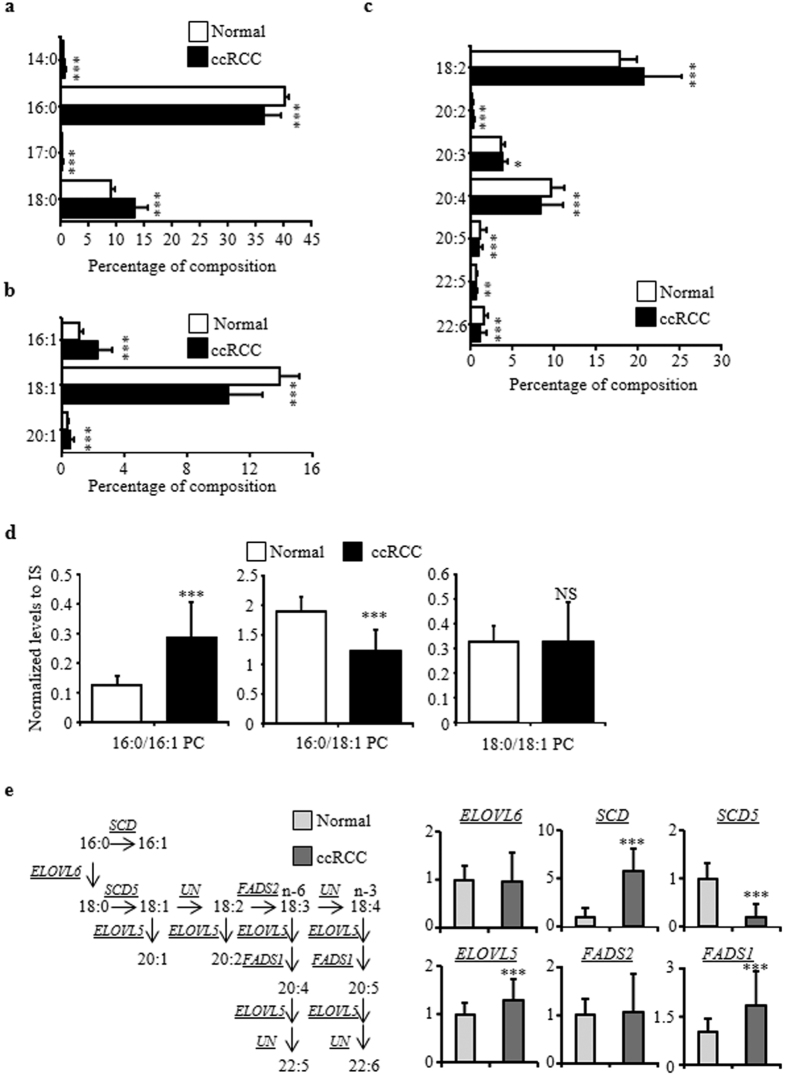
Fatty acid composition of the PC lipids in ccRCC. The abundances of saturated (**a**), monounsaturated (**b**), and polyunsaturated (**c**) fatty acids were calculated as the ratio of the sum of the ion peak heights containing the fatty acid to 2 × the sum of the peak heights of all PC lipids (because one PC lipid contains two fatty acid chains). The data are presented as the means ± SD (*N* = 49 in each group). The statistical significance of the differences between measurements in the cancerous and normal tissues was assessed using a paired Student’s *t*-test with FDR adjustment as follows: **P < 0.01; ***P < 0.001; NS, not statistically significant. (**d**) The levels of 16:0/16:1 PC, 16:0/18:1 PC, and 18:0/18:1 PC are expressed as the means ± SD (*N* = 49 in each group). The statistical significance of the differences between measurements in the cancerous and normal tissues was assessed using a paired Student’s *t*-test with FDR adjustment as follows: ***P < 0.001; NS, not statistically significant. (**e**) Levels of the mRNAs for lipogenic genes that encode proteins involved in fatty acid elongation. The gene with the highest average intensity was selected as the representative of each pathway. The data are presented as relative mean levels ± SD (*N* = 95 in each group), where the levels in the normal tissues were set to 1. The statistical significance of the differences between measurements in the cancerous and normal tissues was assessed using a paired Student’s *t*-test with FDR adjustment as follows: ***P < 0.001; NS, not statistically significant. The abbreviations are defined in Supplementary Table S6.

Our results showed that although the total PC levels were comparable between the normal and cancerous tissues, the levels of more than 70% of the individual PC molecules were significantly different between the two conditions. These results suggest that there would be drastic differences in the combinations of fatty acid side chains in the PC lipids between the normal and cancerous tissues. Thus, we determined the compositions of the PC fatty acid side chains (Supplementary Table S1) and calculated the content of each fatty acid in PCs (Fig. 4a–c). The levels of the major saturated fatty acids (SFAs) (palmitate; 16:0 FA) and monounsaturated fatty acids (MUFAs) (oleate; 18:1 FA) were reduced in the ccRCC samples, whereas the levels of some other SFAs and MUFAs were actually increased in the pathological tissue (Fig. 4a,b). Among the PC molecules, the 16:0/16:1 PC levels were higher, the 16:0/18:1 PC levels were lower, and the 18:0/18:1 PC levels were unchanged in the ccRCC samples (Fig. 4d). On the other hand, the major PUFAs (18:2 FA) and less unsaturated PUFAs (20:2 and 20:3 FAs) were more abundant in the cancerous tissue, whereas the highly unsaturated PUFAs (20:4, 20:5, 22:5 and 22:6 FAs) were present at lower levels (Fig. 4c). The differences in the compositions of the highly unsaturated PUFAs between the normal and cancerous tissues correlated with the differences in the free concentrations of these PUFAs (Fig. 1).

To elucidate the mechanism by which the PC fatty acid composition is altered, we focused on the expression levels of the lipidomic genes that encode proteins involved in fatty acid elongation (Fig. 4e). Expression of the *SCD* gene, which encodes an enzyme involved in 16:0 FA desaturation, was significantly increased and the levels of *SCD5*, which encodes a protein that is necessary for 18:0 FA desaturation, were significantly decreased in the ccRCC samples. These findings were in agreement with the relatively higher levels of 16:1 FA compared to 16:0 FA and lower levels of 18:1FA compared to 18:0 FA that were associated with the pathological changes (Fig. 4a,b). In addition, higher levels of the *ELOVL5* gene, which encodes an enzyme catalyzing the elongation of 18:1 FA to 20:1 FA, were in agreement with the increased levels of 20:1 FA compared to 18:1 FA in the cancerous tissue. On the other hand, the expression of the *FADS1* gene, which encodes an enzyme that synthesizes highly unsaturated PUFAs (20:4, 20:5, 22:5, and 22:6 FA), was increased in the ccRCC samples. Because the levels of highly unsaturated PC-PUFAs and free concentrations of these PUFAs were reduced in the cancerous tissue, the elevated *FADS1* expression level may be a homeostatic reaction to the reduced PUFA levels in ccRCC.

### Comparison of the lipid profiles between low and high grade ccRCC

Because our findings of the lipid alterations in ccRCC were analyzed in all grades of ccRCC together, we also examined the differences in the lipid profiles between low and high grade ccRCC. To clearly discriminate between low and high grade and balance the number of samples, we grouped cancerous tissues with grade 1 or 2 in both the predominant and highest histological grades as low grade and those with grade 3 or 4 in both the predominant and highest histological grades as high grade (N = 16 for low grade and N = 18 high grade). As shown in Supplementary Fig. S2, the levels of all of the lipid classes, except for PE, were constant between low and high grade ccRCC. The levels of the total PE molecules were significantly increased in high grade ccRCC, but the levels were reduced in both grades compared with normal tissue (2.21 ± 0.49 for low grade, 3.33 ± 1.05 for high grade and 7.88 ± 1.77 for normal tissue). In addition, no significant difference was observed in the PC fatty acid composition between low and high grade ccRCC, although the major PUFAs (18:2 FA) tended to be more abundant in low grade ccRCC (p = 0.019). These results suggest that the lipid profiles are relatively constant among the ccRCC grades and that the lipid alterations in ccRCC occurred in the early stage.

### Effect of ethanolamine on the proliferation of renal cancer cells

**Figure 5:**
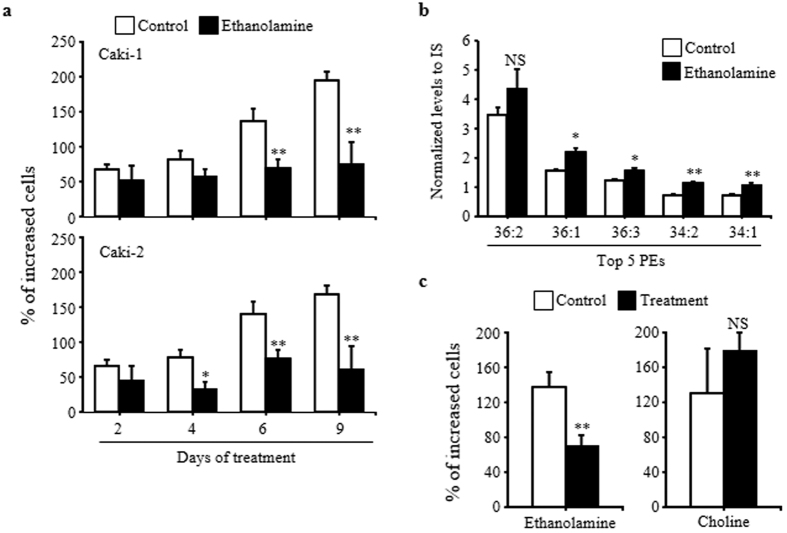
Effects of ethanolamine on the proliferation of cultured renal cancer cells. (**a)** Cell proliferation was determined by the fractional increase in cell numbers after the start of the ethanolamine treatment. The cells were cultured with or without ethanolamine (10 mM) for the indicated number of days. The data are presented as the means ± SD (*N* = 3 in each group). The statistical significance of the differences between measurements in cells cultured with or without ethanolamine was evaluated using Student’s *t*-test as follows: *P < 0.05; **P < 0.01; NS, not statistically significant. (**b**) The levels of 5 major PEs in cultured cells grown in the presence or absence of 10 mM ethanolamine for 4 days were determined as described in the Materials and Methods. The data are presented as the means ± SD (*N* = 3 in each group). The statistical significance of the differences between measurements in cells cultured with or without ethanolamine was evaluated using Student’s *t*-test as follows: *P < 0.05; **P < 0.01; NS, not statistically significant. (**c**) Cell proliferation was determined in the cultures grown in the presence or absence of 10 mM ethanolamine or choline for 6 days. The data are presented as the means ± SD (*N* = 3 in each group). The statistical significance of the differences from vehicle-treated cultures was assessed using Student’s *t*-test as follows: *P < 0.05; NS, not statistically significant.

Because lower PE levels are one of the key features of ccRCC in the present study, we further investigated the impact of components of the PE synthesis pathway on RCC proliferation. We treated the renal cancer cell lines Caki-1 and Caki-2 with ethanolamine to increase their cellular PE levels. As shown in Fig. 5a, ethanolamine treatment inhibited the proliferation of the Caki-1 and Caki-2 cells beginning at day 6 and 4, respectively. Because these effects of ethanolamine were observed in both the Caki-1 and Caki-2 cell lines, we used the Caki-1 cells for the subsequent experiments. To verify that the PE levels were increased by the treatment, we measured the levels of the top 5 PE lipids in Caki-1 cells that were treated with ethanolamine for 4 days, i.e., at a time when the cell numbers in the treated and control groups were not significantly different. As shown in Fig. 5b, the levels of 4 out of the top 5 PE lipids (36:1, 36:3, 34:2, and 34:1 PEs) were significantly increased by the ethanolamine treatment; 36:2 PE showed a similar trend but did not reach statistical significance. Ethanolamine exerted its inhibitory effect on cell proliferation at a concentration of 5 mM, although nominal suppressive action could be observed at a concentration of 2.5 mM (Supplementary Fig. S4). Caki-1 cell viability remained at a level of over 90% in the presence of ethanolamine but was significantly decreased following 6 days of exposure to 10 mM ethanolamine (Supplementary Fig. S5). In addition, incubation with choline, a building block of PC lipids, did not affect Caki-1 cell proliferation (Fig. 5c). Once the inhibitory effect of ethanolamine on the proliferation of renal cancer cell lines was observed, we next examined the effect of ethanolamine on “normal” renal cells (Human renal proximal tubule epithelial cells [RPTEC]). As shown in Supplementary Fig. S6, neither ethanolamine nor choline modulated RPTEC proliferation.

## Discussion

The application of metabolomics to cancer studies expands our understanding of the metabolic alterations that are associated with cancer progression. In the present study, we examined changes in the lipid profiles in cancerous and adjacent normal renal cortex tissue samples obtained from 49 ccRCC patients. In agreement with previous findings^14^,^15^,^16^, our study clearly showed that ChE and TG accumulated in ccRCC tumor tissues. Furthermore, we found several additional characteristic features of ccRCC, namely, (a) lower levels of PE and CL; (b) an altered Cer/SM ratio; (c) higher levels of ePC and ePE lipids; (d) unchanged levels of COX metabolites on the background of lower levels of their substrate PUFAs; and (e) modification of major PC fatty acid side chains.

In the present study, the lower PE lipid content represents a significant feature of ccRCC. In addition, components of the PE synthesis pathway were downregulated at the mRNA level. Moreover, our cell-based assay demonstrated that ethanolamine-induced upregulation of the PE levels inhibited RCC cell proliferation, but not the proliferation of normal renal cells. Thus, we can conclude that PE synthesis is downregulated in ccRCC and hypothesize that this may lead to tumor development by accelerating cell proliferation. The underlying mechanism by which PE abundance regulates the proliferation of renal cancer cells remains to be elucidated. However, PE lipids are known to be exposed on the surface of apoptotic cells^27^. In addition, it has been demonstrated that 16:1/16:1 PE induces apoptosis of malignant mesothelioma cells^28^. In fact, the viability of renal cancer cells enriched in PE was significantly reduced. Thus, one possible mechanism by which the lower PE levels affect cell proliferation in ccRCC might be the inhibition of apoptosis.

In addition to the PE concentrations, we found that the CL level was also reduced in the cancerous tissue. CL is a specific mitochondrial phospholipid and abnormalities in this lipid have been detected in mouse brain tumors^29^. Because mitochondrial dysfunction is a common feature in cancer cells^30^, lower CL levels would be one of the possible mechanisms of mitochondrial dysfunction in ccRCC. It has been reported that mitochondrial CL and PE have overlapping functions in maintaining the tubular morphology of *Saccharomyces cerevisiae*^31^. Although it remains unclear whether changes in the total PE content in ccRCC reflect parallel alterations in the mitochondrial PE levels, the latter could also play a partial role in mitochondrial dysfunction.

In contrast to acyl-phospholipids, ether-phospholipids (ePC and ePE) were present in higher concentrations in the cancerous tissue compared with their levels in the normal renal cortex. In addition, we observed that components of the eDG synthesis pathway were upregulated in ccRCC at the mRNA level. Because ether-phospholipids are also membrane constituents, higher levels of these phospholipids might compensate for a lower content of other phospholipids, such as PE, PI, and CL. Currently, the functional role of ether-phospholipids in ccRCC remains unclear. It has been previously reported that 34:1 and 36:1 ePEs were more abundant in aggressive metastatic breast cancer cells than in cells with a lower degree of aggressiveness^11^. In addition, higher levels of ePC were also detected in migratory breast cancer cells^12^. Thus, ether-phospholipids might be involved in ccRCC metastasis. The first step of ether-phospholipid biosynthesis, unlike that of acyl-phospholipid biosynthesis, occurs in peroxisomes and involves alkylglycerone phosphate synthase (AGPS) and dihydroxyacetone phosphate acyltransferase (GNPAT)^32^,^33^. Because the subsequent steps of ether-phospholipid synthesis are shared with those of acyl-phospholipid synthesis, our results point to the possibility that peroxisomal activity, particularly the expression of AGPS, was enhanced in ccRCC. Recent studies have suggested that the nature of the acyl side chains may alter the ability of ligands to activate PPARα, depending on the tissue. For example, 16:0/18:1 PC activates PPARα in the liver^19^, while 18:0/18:1 PC activates PPARα in muscle^20^. Although the exact composition of the acyl side chains of the PC lipids that activate PPARα in the kidney remains unknown, alterations in the PC fatty acid side chains may be modulated by peroxisomal activity in ccRCC.

SM and Cer can be enzymatically converted to each other. In the present study, the ccRCC tissue samples were characterized by an increased Cer/SM ratio compared with that in the normal surrounding tissue. This alteration could be explained by an upregulation of the SM-to-Cer conversion pathway in the cancerous tissue. It has been reported that cells enriched in SM were more sensitive to apoptosis^34^. In addition, it has also been reported that enriched SM levels are associated with the anticancer effect of 2-hydroxyoleate^35^. Thus, the reduced SM levels observed in the ccRCC samples may promote cancer cell survival.

COX2 is expressed in human renal cell carcinoma tissues^36^,^37^ and is associated with tumor cell proliferation and progression-free survival^37^,^38^. In the present study, free and PC-incorporating PUFAs were present in lower concentrations in the ccRCC samples compared with their levels in the normal renal cortex. In addition, P450 metabolites were also less abundant in the cancerous tissue. In contrast, the levels of COX2 metabolites, such as prostaglandin D_2_, were not different between the normal and pathological samples. These observations suggest that PUFAs were utilized in the ccRCC tissue to produce COX2 metabolites.

Although our study showed that the fatty acid composition of PC was significantly altered in ccRCC, the change was relatively small. However, multiple studies have shown that fatty acid composition is affected during various diseases, such as hepatocellular carcinoma and NASH^39^,^40^. In addition, at least in NASH, the difference in the fatty acid composition is compatible with our present study and is associated with progression from normal to nonalcoholic fatty liver diseases and subsequent NASH. Thus, alteration of the fatty acid composition in ccRCC could be biologically significant, although the role of the fatty acid composition in ccRCC remains unclear.

## Conclusions

In conclusion, our comprehensive lipidomics study using samples of the cancerous and normal renal cortex of ccRCC patients revealed significant changes in the lipid profiles associated with ccRCC. Elucidating the molecular basis of these changes will lead to a better understanding of ccRCC pathophysiology and the development of novel, specific drugs that can be used to treat ccRCC.

## Materials and Methods

### Patients and tissue samples

Forty-nine (for lipidomics) and ninety-five (for transcriptome analysis) paired samples of the cancerous tissue and surrounding normal renal cortex were obtained from patients with primary ccRCC. The patients had not received any preoperative treatment and had undergone nephrectomy at the National Cancer Center Hospital (Tokyo, Japan). Histological diagnosis was made in accordance with the World Health Organization classification^41^. All tumors were graded on the basis of previously described criteria^42^ and classified according to the pathological tumor-node-metastasis (TNM) classification^43^. The clinicopathological parameters of the patients whose samples were used in the lipidomic and transcriptome analyses are summarized in Table 2.

All patients included in this study provided written informed consent. This study was approved by the Ethics Committees of the National Cancer Center and the National Institute of Health Sciences and was performed in accordance with the Declaration of Helsinki.

### Lipid extraction

The lipids were extracted from the tissues as previously described^18^. In brief, the lipids were extracted from 9 mg of tissue using the Bligh and Dyer method, with several modifications. The lower phase (corresponding to 20 μg of tissue) was used to measure the phospholipid, sphingolipid, neutral lipid, and acylcarnitine levels. The upper phase (corresponding to 3 mg of tissue) was subjected to solid extraction and was used to measure the levels of PUFAs and their metabolites. Hexadeuterated 16:0/16:0 PC (16:0/16:0-d6 PC; Larodan Fine Chemicals, Malmö, Sweden), 8:0/8:0/18:2 TG (Larodan Fine Chemicals), and tetradeuterated leukotriene B_4_ (LTB_4_-d4; Cayman Chemical, Ann Arbor, MI) were added as internal standards before extraction.

### Non-targeted measurements of the phospholipid, sphingolipid, neutral lipid, and acylcarnitine levels

The phospholipid, sphingolipid, neutral lipid, and acylcarnitine levels were measured using liquid chromatography-time-of-flight mass spectrometry (LC-TOFMS; ACQUITY UPLC System [Waters, Milford]-LCT Premier XE [Waters, Milford]) as previously described^44^. The samples of the cancerous tissue and surrounding normal renal cortex were randomized across the runs. The raw LC-TOFMS data were processed using 2DICAL software (Mitsui Knowledge Industry, Tokyo, Japan), which allows the detection and alignment of the ion peaks for each ionized biomolecule obtained at a specific mass-to-charge ratio (m/z) and column retention time (RT). The main parameters of 2DICAL were set as previously described^44^, with several modifications. To extract the ion peaks of most phospholipids (lysophosphatidylcholine, lysophosphatidylethanolamine, PC, ePC, PE, ePE, PI, and phosphatidylglycerol/bismonophosphotidate) and sphingolipids (SM, Cer, glycosylceramide, and Sul), the RT range was from 2.0 to 38.0 min in the negative ion mode. For the ion peaks of CL, neutral lipids (Ch/ChE, DG, TG, and coenzyme Q), and acylcarnitines, the RT range was from 2.0 to 60.0 min in the positive ion mode. The lipid molecules were identified using the extracted ion peaks by comparing the ion features (RT, m/z, preferred adducts, and in-source fragments) of the experimental samples with those of our reference library of lipid molecule entries, as previously described^44^. Processing of the extracted ion peaks yielded 288 lipid molecules (155 and 133 lipid molecules from the negative and positive ion modes, respectively; Table 1 and Supplementary Table S2).

### Targeted measurements of the levels of PUFAs and their metabolites

The levels of PUFAs and their COX, LOX, and P450 metabolites were measured by the targeted approach using the LC-MS/MS (ACQUITY UPLC System-5500 QTRAP quadrupole-linear ion trap hybrid mass spectrometer [AB Sciex, Framingham, MA]), as previously described^44^. Samples of the cancerous tissue and surrounding normal cortex were randomized across the runs. The targeted lipid molecules were annotated by comparing their RT, parent ion, and MS/MS ion fragment data with those of standard lipid molecules using MultiQuant Software (Version 2.1, AB Sciex). Processing of targeted lipid molecules yielded 38 individual substances (Table 1 and Supplementary Table S2).

### Lipidomic data processing

The data cutoff point of non-targeted measurements was set at 50 for the negative ion mode and 100 for the positive ion mode for height, while the cutoff point of targeted measurements was set at 10 for the signal-to-noise ratio. For samples with missing values for a metabolite, a cutoff point of 50 (negative ion mode) or 100 (positive ion mode) was applied to the non-targeted measurements, and the minimum observed value of the metabolite among all samples was used in the targeted measurements. Because this study spanned multiple run sequences, the median value of each metabolite in each run sequence was normalized to its mean value to correct for the variability resulting from instrument inter-run tuning differences. In addition, to correct for the variability of each sample run, the intensities of each extracted ion peak were normalized to those of the internal standard (16:0/16:0-d6 PC for phospholipids and sphingolipids, and 8:0/8:0/18:2 TG for neutral lipids and acylcarnitines) in the non-targeted measurements. For the targeted measurements, the areas of each ion peak of the targeted lipid molecules were normalized to those of the internal standard (LTB_4_-d4). The values of the relative standard deviation of the internal standard (16:0/16:0-d6 PC, 8:0/8:0/18:2 TG, and LTB_4_-d4) were monitored for experimental quality control throughout the extraction, measurement, and data processing and were 6.3%, 8.4%, and 24.3%, respectively. All data obtained from the non-targeted measurements were presented as relative fractions of the internal standard levels, whereas those obtained from the targeted measurements were presented as values normalized to the median values of all measured samples. Significant differences in metabolite levels were assessed by paired Student’s *t*-test. The correction for multiple comparisons in the lipidomic analysis was performed by calculating the FDR using the Benjamini and Hochberg method^45^. In this study, the lipidomic measurements were considered to be significantly different if *P* < 0.01. The processed data, average values, and standard deviations of the lipid molecule levels, as well as he statistical information (raw and corrected *P* values), are presented in Supplementary Table S3. For comparison between low and high grade ccRCC, the average values and standard deviations of the lipid molecule levels, as well as statistical information (raw and corrected *P* values), are presented in Supplementary Table S4.

### Transcriptome analysis

RNA was extracted from snap-frozen tissue samples using TRizol reagent (Thermo Fisher Scientific, Waltham, MA). The quality and quantity of the extracted RNA molecules were examined using a Bioanalyzer (Agilent Technologies, Santa Clara, CA). The threshold for the RNA integrity number was set at 0.6. A SurePrint G3 Human Gene Expression v3 8 × 60 K Microarray Kit (Agilent Technologies) was used to determine expression levels of the lipogenic genes. The raw data were processed using quantile normalization, and statistical comparisons were performed with the paired Student’s *t*-test. The normalized expression data were deposited in the Integrative disease Omics Database (http://gemdbj.ncc.go.jp/omics/) (accession No. EXPR059). Because multiple comparisons were performed in the microarray analysis, the *P* values from the statistical tests were corrected using the Bonferroni adjustment. Mapping of the lipogenic genes to selected reaction pathways and fatty acid elongation was performed using the KEGG pathway database (www.genome.jp/kegg/pathway) as the reference. The processed data for the mapped genes are listed in Supplementary Tables S5 (lipidomic network) and S6 (fatty acid elongation).

### Cell-based assays

The RCC Caki-1 (cell number: JCRB0801) cell line was obtained from the JCRB Cell Bank (Osaka, Japan). Another RCC cell line, Caki-2, was purchased from DS Pharma Biomedical (Osaka, Japan). The RPTEC were purchased from Lonza (Walkersville, MD). Caki-1 and Caki-2 cells were maintained in advanced MEM (Thermo Fisher Scientific) supplemented with 1% (vol/vol) charcoal-stripped fetal bovine serum (Cosmo Bio Co., Ltd., Tokyo, Japan), antibiotics (100 U/mL penicillin and 100 μg/mL streptomycin), and 1 × GlutaMAX (Thermo Fisher Scientific). Caki-1 and Caki-2 cells (5 × 10^4^ cells/mL) were plated on 12-well plates (for cell proliferation assays) or 6-well plates (for the determination of PE levels), incubated for 24 h, and subjected to serum-free media changes and chemical treatments. Serum-free medium was prepared by substituting serum with 1 × ITS Liquid Media Supplement (Thermo Fisher Scientific). RPTEC were maintained in MEGM, Mammary Epithelial Cell Growth Medium, (Lonza). RPTEC (2 × 10^4^ cells/mL) were plated on 12-well plates for the cell proliferation assay, incubated for 24 h, and subjected to chemical treatments. For the treatment, the cells were exposed to different concentrations of ethanolamine chloride, choline hydrochloride, or vehicle control (water) for the indicated number of days.

For the cell proliferation assays, the cells were collected and counted by a Scepter Cell Counter (EMD Millipore, Billerica, MA) after the indicated number of treatment days. Cell proliferation was presented as a percent increase in the number of cells on the indicated days of treatment compared with the cell numbers before chemical treatment. The extent of cell proliferation was presented as percentage of relative increase compared with the basal cell numbers. To determine the PE levels, the cells from one well of a 6-well plate were collected by scraping with PBS, centrifuged, and resuspended with methanol containing 12:0/12:0 PE (internal standard). Subsequently, the cells were thoroughly vortexed and centrifuged to remove the cellular debris. The resulting supernatant was subjected to non-targeted measurements to determine the PE levels, as described above.

## Additional Information

**How to cite this article**: Saito, K. *et al*. Lipidomic Signatures and Associated Transcriptomic Profiles of Clear Cell Renal Cell Carcinoma. *Sci. Rep.* **6**, 28932; doi: 10.1038/srep28932 (2016).

## Supplementary Material

Supplementary Information

Supplementary Information

## Figures and Tables

**Table 1:** Identified lipid classes and numbers of individual lipid molecules in ccRCC.

Lipid type	Lipid classes	Number of molecules
Phospholipid	lysophosphatidylcholine (LPC)	2
	lysophosphatidylethanolamine (LPE)	2
	phosphatidylcholine (PC)	25
	ether-type PC (ePC)	13
	phosphatidylethanolamine (PE)	25
	ether-type PE (ePE)	23
	phosphatidylinositol (PI)	14
	phosphatidylglycerol/bismonophosphotidate (PG/BMP)	4
	cardiolipin (CL)	9
Sphingolipid	sphingomyelin (SM)	13
	ceramide (Cer)	8
	glycosylceramide (GCer)	14
	Sulfatide (Sul)	12
Neutral lipid	coenzyme Q (CoQ)	1
	cholesterol/cholesterol ester (Ch/ChE)	25
	diacylglycerol (DG)	10
	triacylglycerol (TG)	76
PUFA and their metabolites	Parents	3
	cyclooxygenase (COX) metabolites	6
	lipoxygenase (LOX) metabolites	15
	cytochrome P450 (P450) metabolites	14
Acylcarnitine	acylcarnitine (Car)	12
	total	326

**Table 2:** Clinicopathological parameters of ccRCC.

Factors		for lipidomics	for transcriptome analysis
Age		64.80 ± 11.12	62.87 ± 10.56
		(range 38 to 87)	(range 36 to 85)
Sex	Male	39	66
	Female	10	29
Tumor diameter (cm)		6.14 ± 3.11	5.85 ± 3.35
		(range 1.6 to 16.5)	(range 1.5 to 16.0)
Macroscopic configuration	Type 1	11	31
	Type 2	17	29
	Type 3	20	35
	other^a^	1	0
Predominant histological grades^b^	Grade 1	9	44
	Grade 2	22	34
	Grade 3	14	14
	Grade 4	4	3
Highest histological grades^c^	Grade 1	3	7
	Grade 2	13	39
	Grade 3	22	25
	Grade 4	11	24
Vascular involvement	Negative	14	47
	Positive	35	48
Renal vein tumor thrombi	Negative	29	66
	Positive	20	29
Predominant growth pattern^b^	Expansive	31	82
	Infiltrative	18	13
Most aggressive growth pattern^c^	Expansive	26	56
	Infiltrative	23	39
Tumor necrosis	Negative	28	64
	Positive	21	31
Invasion to renal pelvis	Negative	40	85
	Positive	9	10
Pathological TNM stage	Stage I	22	43
	Stage II	1	4
	Stage III	14	25
	Stage IV	12	23
Total		49	95

^a^Cystic.

^b^If the tumor showed heterogeneity, the findings in the predominant area were described.

^c^If the tumor showed heterogeneity, the most aggressive features of the tumor were described.
