# Supplementary material for: Lipidomic Signatures and Associated Transcriptomic Profiles of Clear Cell Renal Cell Carcinoma
Source: Sci Rep. 2016 Jun 30;6:28932. doi: 10.1038/srep28932 (PMC4928052; doi:10.1038/srep28932)
Supplement: Supplementary Information [file srep28932-s1.pdf]

Supplemental figures

Lipidomic Signatures and Associated Transcriptomic Profiles of Clear Cell Renal Cell Carcinoma

Kosuke Saito[1#], Eri Arai[2,3#], Keiko Maekawa[1], Masaki Ishikawa[1], Hiroyuki Fujimoto[4], Ryo Taguchi[1], Kenji Matsumoto[5], Yae Kanai[2,3*] & Yoshiro Saito[1*]

Supplementary Figure S1

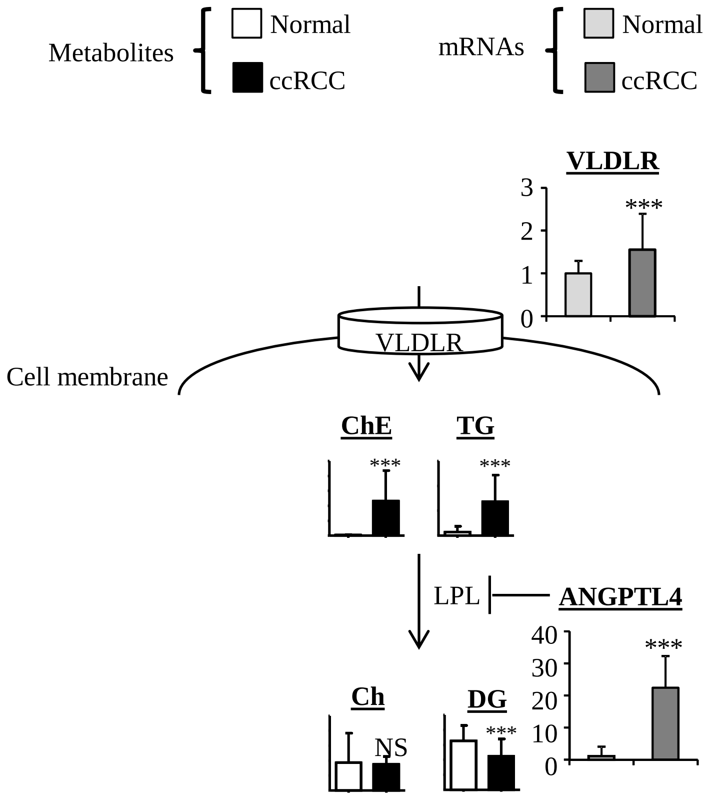

Supplementary Figure S1. Levels of *VLDLR* and *ANGPTL4* mRNA expression in ChE and TG pathway. The gene with the highest average intensity was selected as the representative of each pathway. Data are presented as the mean relative levels ± SD (N = 95 in each group) when levels in normal tissues were set as 1. Statistical significance of differences between measurements in the cancerous and normal tissues was assessed by the Student's paired t-test with the FDR adjustment and shown as follows: ***, $P < 0.001$; NS, not statistically significant. Abbreviations were described in Table 1.

Supplementary Figure S2

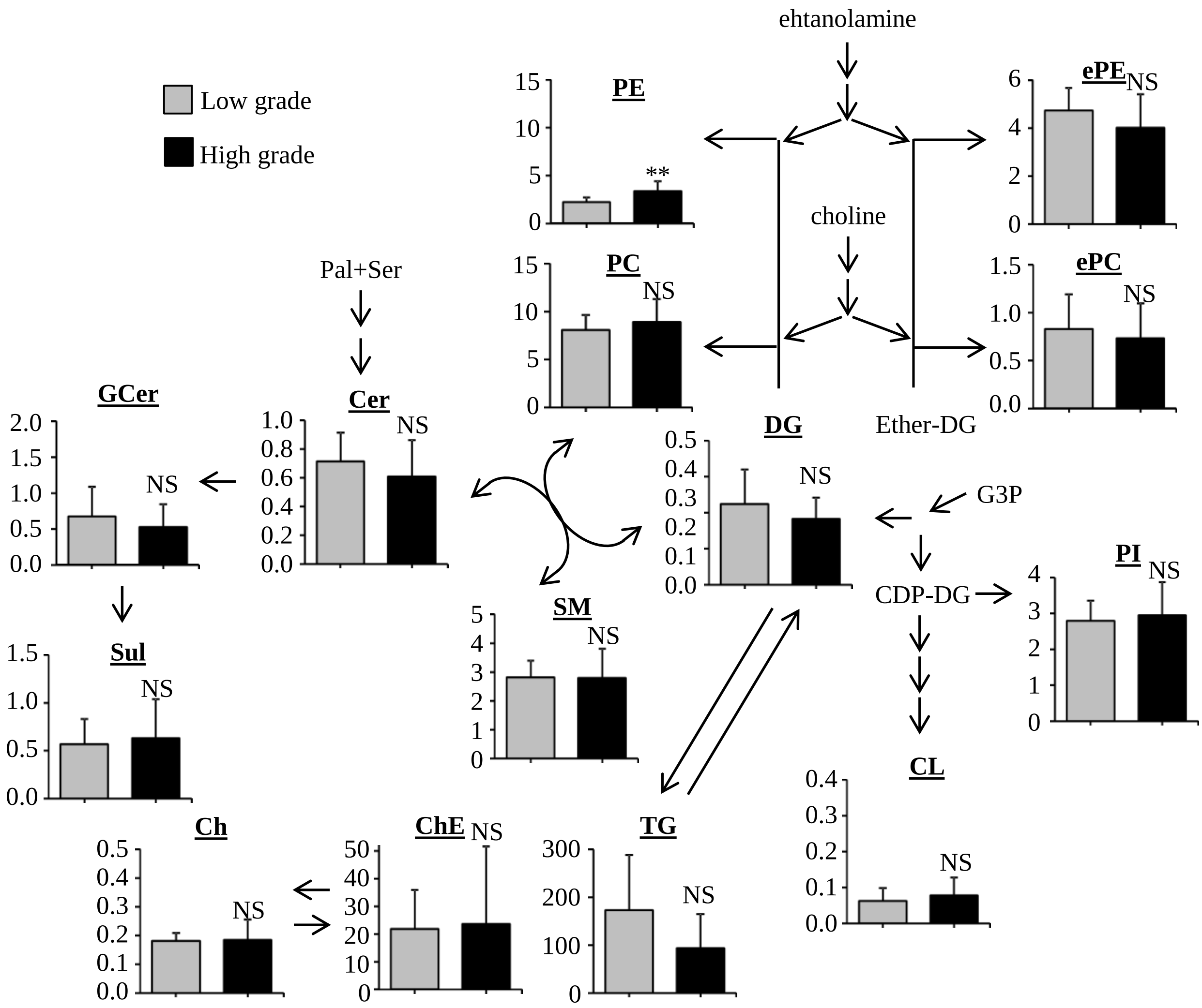

Supplementary Figure S2. Lipidomic network in low (grade 1 and 2) and high grade (grade 3 and 4) ccRCC. Data represent sums of ion peak heights of all lipid molecules within each class and are illustrated as the mean ± SD (N =16 for low grade and N=18 for high grade). Statistical significance of differences between measurements in the tissues of low and high grade ccRCC was assessed by the Welch’s t-test with FDR adjustment and is shown as follows: **, $P < 0.01$; NS, not statistically significant. Abbreviations were described in Table 1.

Supplementary Figure S3

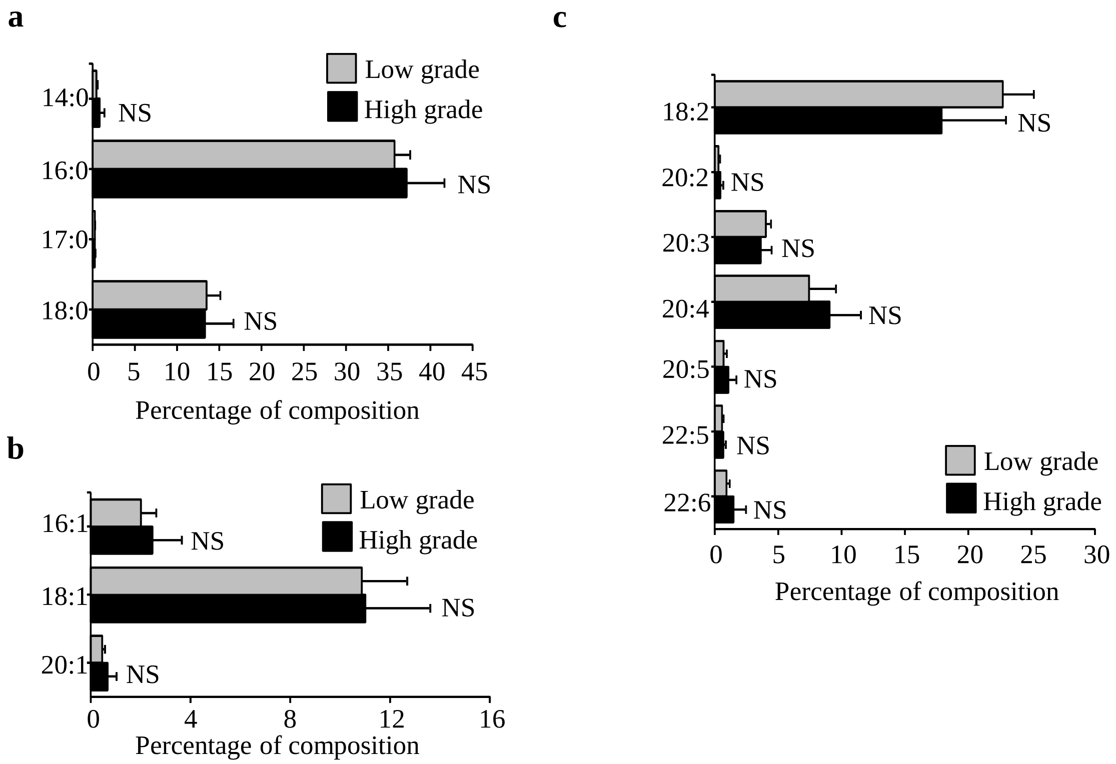

Supplementary Figure S3. Fatty acid composition of PC lipids in low (grade 1 and 2) and high grade (grade 3 and 4) ccRCC. Abundance of saturated (a), monounsaturated (b), and polyunsaturated (c) fatty acids was calculated as the ratio of the sum of ion peak heights containing the fatty acid to 2 $\times$ sum of peak heights of all PC lipids (because one PC lipid contains two fatty acid chains). Data are presented as the mean $\pm$ SD (N =16 for low grade and N=18 for high grade). Statistical significance of differences between measurements in the tissues of low and high grade ccRCC was assessed by the Welch's t-test with FDR adjustment and is shown as follows: NS, not statistically significant.

Supplementary Figure S4

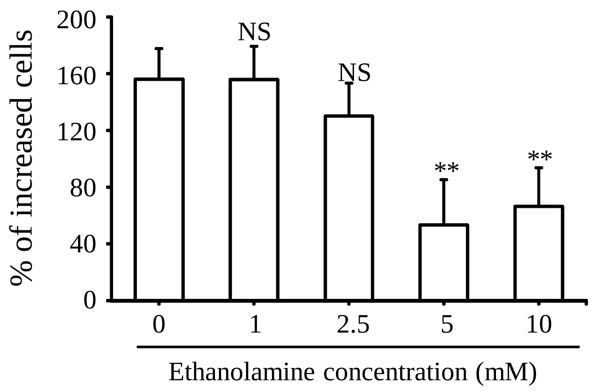

Supplementary Figure S4. Dose-dependent effects of ethanolamine on the proliferation of Caki-1 cells. Cell proliferation was determined by increase in cell numbers after the start of the ethanolamine treatment. Cells were cultured with indicated concentrations of ethanolamine for 6 days. Data are presented as the mean ± SD (*N* = 3 in each group). Statistical significance of differences between measurements in cells cultured with indicated concentrations of ethanolamine was assessed by one-way ANOVA followed by *t*-test and indicated as follows: **, $P < 0.01$; NS, not statistically significant.

Supplementary Figure S5

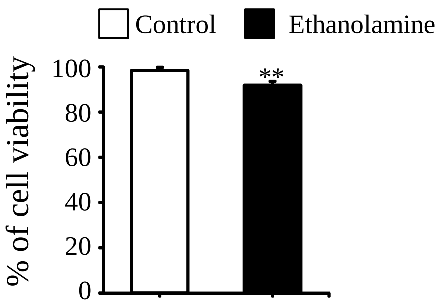

Supplementary Figure S5. Effects of ethanolamine on the cell viability of Caki-1 cells. Cells were cultured in the presence or absence of 10 mM ethanolamine for 6 days and determined cell viability. Data are presented as the mean ± SD (*N* = 3 in each group). Statistical significance of differences between measurements in cells cultured in the presence or absence of 10 mM ethanolamine was assessed by Student's *t*-test and indicated as follows: **, *P* < 0.01.

Supplementary Figure S6

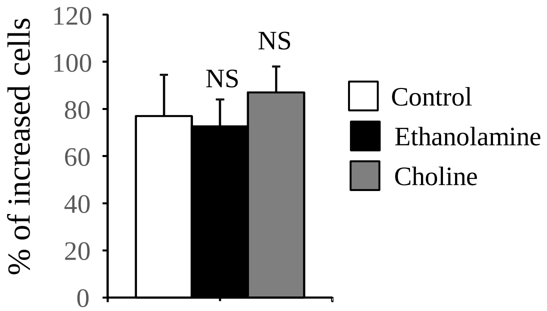

Supplementary Figure S6. Effects of ethanolamine on the proliferation of cultured normal primary renal tubular epithelial cells. Cell proliferation was determined in cultures grown in the presence or absence of 10 mM ethanolamine or choline for 6 days. Data are presented as the mean ± SD (N = 4 in each group). Statistical significance of differences from vehicle-treated cultures was assessed by the Student’s t-test and indicated as follows: NS, not statistically significant.
